# Diffuse optical tomography system for acute traumatic brain injury in the intensive care unit: a prospective study on healthy volunteers

**DOI:** 10.1117/1.JBO.30.S2.S23912

**Published:** 2025-09-17

**Authors:** Mario Forcione, Antonio Maria Chiarelli, David Perpetuini, Guy A. Perkins, Andrew R. Stevens, David J. Davies, Antonio Belli

**Affiliations:** a University of Birmingham, Institute of Inflammation & Ageing, College of Medical and Dental Sciences, Neuroscience & Ophthalmology Research Group, Birmingham, United Kingdom; b University of Milan-Bicocca, School Medicine and Surgery, Milan, Italy; c University “G. D’Annunzio” of Chieti-Pescara, Institute for Advanced Biomedical Technologies (I.T.A.B.), Department of Neuroscience, Imaging, and Clinical Sciences, Chieti, Italy; d University of Birmingham, College of Engineering and Physical Sciences, School of Computer Science, Birmingham, United Kingdom; e University of Padua, Department of Developmental Psychology and Socialisation, Padua, Italy; f University Hospitals Birmingham NHS Foundation Trust, Neurosurgery Department, Birmingham, United Kingdom

**Keywords:** diffuse optical tomography, functional near-infrared spectroscopy, Valsalva maneuver, traumatic brain injury, intensive care unit

## Abstract

**Significance:**

Invasive neuromonitoring hinders the application of diffuse optical tomography (DOT) to critically ill adults in the intensive care unit (ICU).

**Aim:**

We aim to develop and test a method for DOT recordings suitable for traumatic brain injury (TBI) patients in the ICU. This method is based on measurements and coregistration using a 3D optical scan and the acquisition of optical data using a custom-made helmet, which would enable a multimodal (invasive and noninvasive) neuromonitoring.

**Approach:**

Coregistration accuracy between the method based on a 3D optical scan and one based on an electromagnetic digitization, the latter considered to be the gold standard, was assessed. The capacity to isolate and monitor, using functional near-infrared spectroscopy, the optical signal in the intracranial (ICT), and extracranial tissues (ECT), was tested on 23 healthy volunteers. Participants were scanned with a frequency-domain NIRS device (690 and 830 nm) during 5 Valsalva maneuvers (VM) in a simulated ICU environment.

**Results:**

The results showed an average error of coregistration of 5.5 mm and a sufficient capacity to isolate oxyhemoglobin ($O_{2}\mathrm{Hb}$) ($p=6.4\cdot 10^{-6}$) and total hemoglobin (HbT) ($p=2.8\cdot 10^{-5}$) in the ICT from the ECT and to follow the changes of hemoglobin in the ICT during the VM ($O_{2}\mathrm{Hb}$, $p=9.2\cdot 10^{-4}$; HbT, $p=1.0\cdot 10^{-3}$).

**Conclusion:**

The developed approach appears to be suitable for use on TBI patients in the ICU in a multimodal monitoring.

## 1 Introduction

Traumatic brain injury (TBI) is a leading cause of injury-related death and disability, resulting in an estimated one million deaths per year worldwide, devastating impacts for survivors and their families, and an estimated cost for society of up to $445 billion/year.^1^ Currently, there is no disease-modifying treatment for the primary injury sustained by the brain during trauma, and the treatment for severe TBI remains limited to the prevention of subsequent insults [e.g., intracranial hypertension, ischemia, hemorragic progression of contusion (HPC)], known as secondary brain injury. Pivotal components of established treatment strategies in TBI patients are continuous neuromonitoring and adaptation of the physiological parameters accordingly to maintain intracranial homeostasis.

Functional near-infrared spectroscopy (fNIRS) represents a noninvasive, continuous neuromonitoring tool for tracking changes in brain oxygenation in severe TBI patients by measuring changes of oxyhemoglobin ($O_{2}\mathrm{Hb}$) and deoxyhemoglobin (HHb) concentrations.^2^^–^^4^ Contrast-enhanced NIRS can also be employed to detect changes in optical density during a dye passage [e.g., indocyanine green (ICG)], allowing one to assess cerebral perfusion and blood-brain barrier (BBB) damage.^5^^–^^7^ So far, commercially available NIRS devices (e.g., INVOS 5100 Cerebral Oximeter; ISS Oxiplex TS) have not shown sufficient ability to detect episodes of ischemia in cases of severe TBI compared with the invasive intracranial techniques [i.e., brain partial oxygen pressure (${\mathrm{PbtO}}_{2}$)].^8^^–^^10^ However, the validity of a direct comparison between (i) the level of cerebral tissue saturation and its fluctuations measured by the optical device and (ii) the absolute values and their changes measured by the ${\mathrm{PbtO}}_{2}$ monitor, can be impaired by the complex pathogenesis of brain trauma.^11^ By contrast, the addition of fNIRS and contrast-enhanced NIRS to currently used neuromonitoring techniques, including ${\mathrm{PbtO}}_{2}$, could potentially lead to a more precise assessment of the tissue status.^5^^,^^11^ Thereby, the drive for “noninvasive equivalence” to ${\mathrm{PbtO}}_{2}$ should be dropped, and instead, the hitherto untested abilities of the optical technique, when included in a multimodal monitoring regime, to stratify injury severity and aid clinical decision-making, could finally be explored.

Diffuse optical tomography (DOT) is an extension of NIRS imaging that uses a distributed high-density array of NIRS probes to spatially map quantitative tissue optical properties (when nonlinear iterative inversion approaches are used) or changes in optical properties (when linear inversion algorithms are used and assuming small changes) on a co-registered structural image [e.g., computerized tomography (CT), magnetic resonance imaging (MRI)].^12^^–^^14^ Compared with commercially available NIRS devices, DOT offers several advantages in the TBI monitoring by addressing the anatomical complexity of the TBI lesions.^11^ This advantage suggests that the commercially available NIRS devices used in clinical trials to date have not fully explored the capabilities of optical techniques and that DOT should be tested instead. Although DOT has been successfully demonstrated as a neuromonitoring technique in infants admitted to the neonatal intensive care unit (ICU), the authors are not aware of any studies, which have performed DOT measurements on adult severe TBI patients in the ICU.^15^^,^^16^ One of the difficulties in performing such studies is related to the clinical environment in which the optical data should be acquired [e.g., contamination of the signal from the ambient light, lack of space at the patient’s bedside, the presence of an intracranial bolt for intracranial pressure (ICP) neuromonitoring]. Furthermore, the standard processes of probe digitization using an electromagnetic digitizer, or of adding skin markers to the subject’s structural imaging, would be impractical in the clinical practice of brain trauma care.^17^^–^^19^ The former can be impaired by the presence of metal at patients’ bedsides (e.g., ventilator, frame of bed, pumps).^20^ With respect to the latter process of digitization, the optical analysis can only be performed after skin markers have been added. Because a CT scan is one of the first neuroimaging assessments made on TBI patients upon hospital admission, it is usually performed urgently;^21^ this makes it unlikely that skin markers could be positioned on TBI patients, at the locations where the optodes will be placed, prior to their first scan. Furthermore, it should be taken into consideration that the CT scan is crucial to the clinical decision process, and the presence of skin markers may increase the risk of imaging artifacts, which can impair accurate radiological assessment. Therefore, a method that co-registers the optical data into a CT scan without the aid of skin markers, or electromagnetic digitization, is needed to make DOT analysis on TBI patients possible from the point of admission.

We devised a system to perform DOT recordings on acute TBI patients from the point of admission into ICU, comprised of a custom-made helmet for optical data acquisition in a multimodal neuromonitoring (e.g., intracranial probes), and a coregistration of the optical data on patients’ structural images acquired for clinical assessment (e.g., CT scans). On healthy volunteers, we tested (i) the accuracy of the novel coregistration compared with one based on electromagnetic digitization and (ii) the capacity to accurately detect changes in levels of Hb in the intracranial tissues (ICT) and separate signals from ICT and extracranial tissues (ECT) in a simulated ICU scenario.

## 2 Materials and Methods

### 2.1 Participants

Twenty-three healthy volunteers (17 males, ages ranging between 20 and 42 years old with a mean of 28 years) were recruited into the RECOS study (Repetitive Concussion in Sport) (IRAS ID: 216703; Ref. REC: 17/EE/0275) performed at Center for Human Brain Health, University of Birmingham, UK. They had different hair colors and hair densities.

Participants took part in this study after providing their written, informed consent. The study was conformed to the Declaration of Helsinki and was approved by the East of England-Essex Research Ethics Committee on September 22, 2017.

### 2.2 Equipment

#### 2.2.1 Optical helmet

**Fig. 1:**
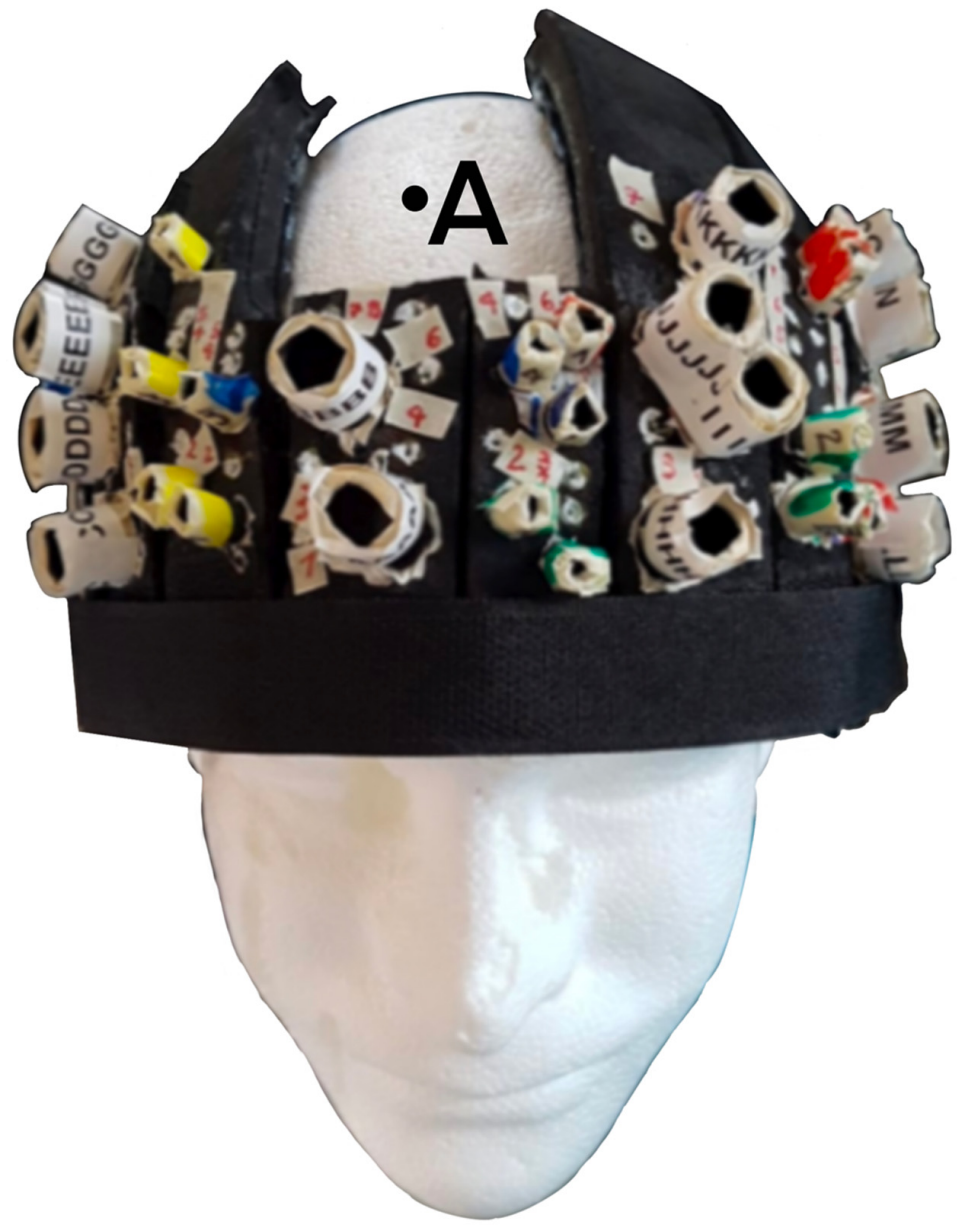
Picture of the helmet. The probe housings for detectors were labeled with letters, and those for sources were numbered and color-coded (yellow, blue, green, and red). The detectors and the sources of the optical system (Imagent; ISS Inc.), not shown here, had a matching labeling. Additional reference points (i.e., white dots) were numbered on the helmet surface. The order of the numbers was chosen to allow one to perform a continuous electromagnetic digitization for each row considering probes and white dots together. The design features an open area at the top of the skull to avoid interference with invasive neuromonitoring devices positioned in the Kocher’s point (estimated at point A) and tunneled a couple of centimeters into the scalp.

A custom-built optical helmet was adapted from the description of Tan et al.^22^ to make it suitable for optical recordings on TBI patients in the ICU (Fig. 1).

The helmet comprised multiple adjustable segments. It could be adjusted to fit a variety of head shapes and sizes, including post-surgery [e.g., decompressive craniectomies, external ventricular drains (EVD)]. It was vital that the helmet design maintained patient comfort and safety (e.g., no increase in ICP) while also being rigid enough to keep the probes still during the recording. The helmet is designed to be affixed to the patient using disposable Velcro straps or medical bandages under the chin. To reduce the risk of infection and to grant access for intracranial monitoring and treatment, no slices were positioned within approximately a 2-cm radius of the Kocher’s point, where the ICP bolt and the EVD are usually positioned and the catheter is tunneled into the scalp [Fig. 1(A)].^21^

The helmet comprised multiple black layers. The thicker part was made of two layers of black foam (The Michaels Companies, Las Colinas, Irving, Texas, USA) to shield the probes from ambient light as well as to keep the helmet lightweight. These two layers were glued together while held in a curved shape, to model the helmet frame. The internal lining of the helmet was made of a thin layer of polyurethane rubber, RenCast (Freeman manufacturing and Supply Company, Avon, Ohio, USA), which came into contact with the scalp, along with the probes. Polyurethane rubber has been used in previous studies on TBI patients in the ICU and, in our experience, it increases the helmet’s integrity whilst also being suitable for clinical environments, as it is nonporous, soft, and easy to clean.^8^^,^^23^

**Fig. 2:**
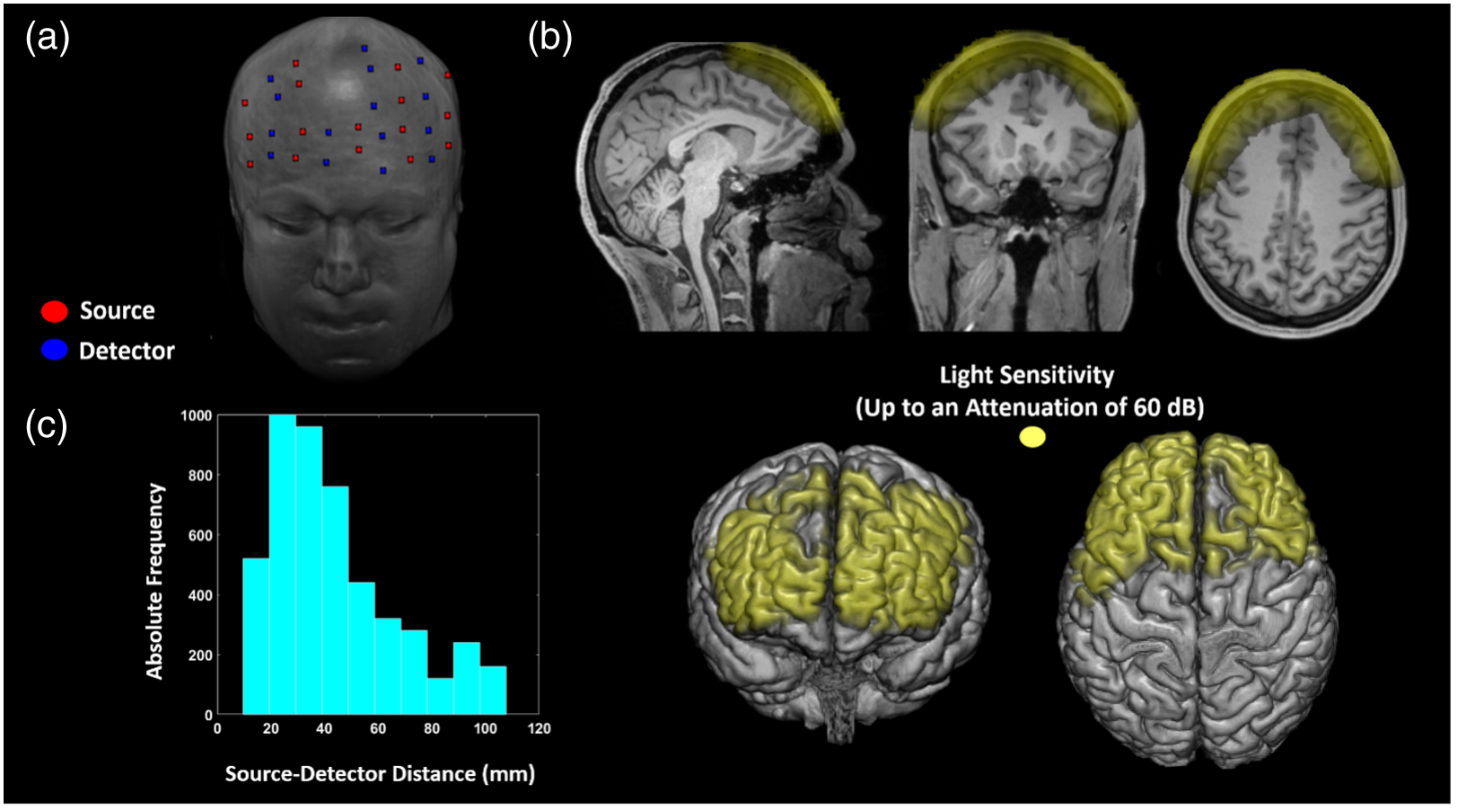
(a) Illustration of the optical probes’ positions on a structural MRI. The sources’ positions are illustrated as red dots and the detectors’ positions as blue dots. (b) Average light sensitivity of the probe configuration used, computed with a FEM approach.^24^ The light sensitivity is displayed up to an attenuation of 60 dB (1000 times), and it is overlaid on an MRI of a representative participant. (c) The total number (i.e., absolute frequency) of the SD distances of the channels across all subjects.

The layout for optical recording covered only the frontal lobes to facilitate the positioning of the probes on TBI patients who have limited head movement due to being sedated in a supine position with their head being tilted-up, as per ICP control care, potentially wearing a hard cervical collar, and being intubated or with a tracheostomy [Fig. 2(a)].^21^^,^^25^

Prior to making holes to house the probes, to decide where they should be located, a “3-space” FastTrak 3-D digitizer (Polhemus, Colchester, Vermont, USA) was used to electromagnetically digitize the helmet surface using the nasion and the external acoustic meatuses (EAM) of an artificial head as fiducial points. Based on the digitization obtained, a distribution of sources and detectors was selected, with the aim to scan the broadest possible area with the maximal number of source-detector (SD) distances between 25 mm and 35 mm, thereby maximizing sensitivity to the ICT [Fig. 2(c)].^12^ A custom-made MATLAB graphical user interface, near-infrared optode montage automated design (NOMAD) (https://github.com/kylemath/nomad), developed by Mathewson et al., was used to evaluate the acceptability of a plotted configuration of SD locations on the helmet, by ensuring the illumination of the frontal lobes of both hemispheres while avoiding cross-talk between SD pairs in a time-multiplexing cycle of light-emission.^26^ The average light sensitivity of the probe configuration used was computed with a FEM approach [Fig. 2(b)].^24^ Below 60 mm distances, the probes were deemed to be usable for fNIRS-DOT analysis based on the data recorded during the Valsalva maneuver (VM). Holes to position the probes were punched only where the NOMAD analysis recommended. The limited number of holes, compared with the model described by Tan et al.,^2^ was necessary to reduce the amount of ambient light that reaches the probes, by ensuring that no hole went unutilized, as well as to allow data recording in a supine position. The large diameter of the holes where the detectors were inserted allowed for the removal of any hair between the detector and the skin, to maximize photon harvesting from the scalp.^27^^,^^28^ A plastic cone was inserted inside the hole along with each detector to mechanically secure them to the helmet and to reduce the signal contamination by ambient light.^28^ The holes were labeled and color-coded to facilitate the positioning of the probes. Small additional reference points, so-called white dots, that could be easily seen on an optical 3D scanner, were drawn on the helmet in the regions that were to be exposed in a supine participant. The number of white dots was chosen arbitrarily for the purpose of a pilot assessment, by considering the practical upper limit given the surface area to be scanned.

### 2.3 Data Acquisition

#### 2.3.1 Digitization

**Fig. 3:**
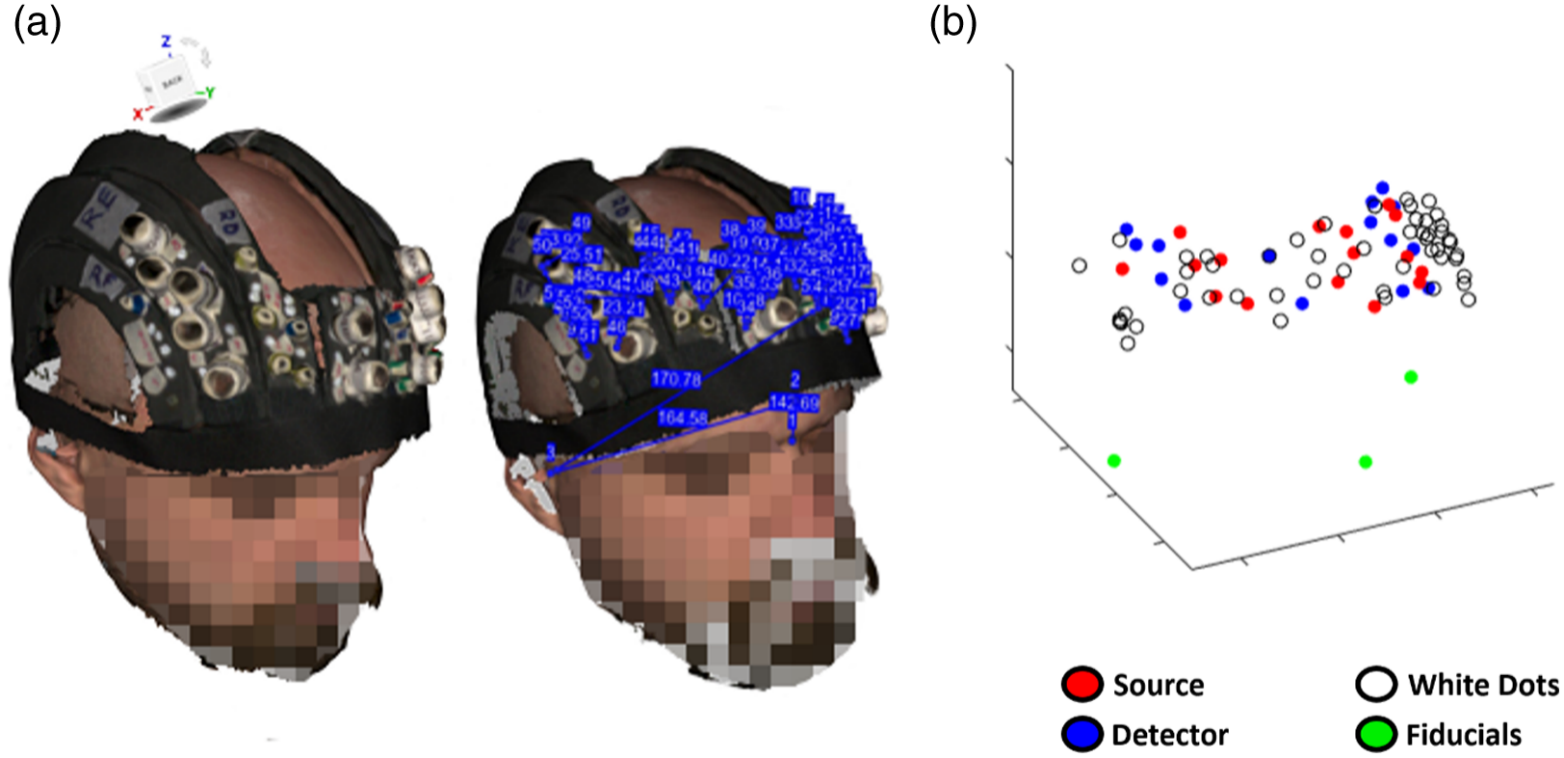
(a) Picture from the optical 3D scanner (Artec Leo) reconstructed using Artec Scanner and measurement of the 3D coordinates of the nasion, EAM, and white dots. (b) Example electromagnetic digitization of fiducials (nasion and EAM), white dots and optodes with FastTrak.

Participants were scanned with an optical 3D scanner (Artec Leo, Artec 3D, Luxemburg, European Union) while wearing the helmet and sitting on a chair. The quality of signal acquisition was confirmed by a visual inspection on the 3D scanner screen. On a 3D image, the x−y−z coordinates of the fiducials (i.e., nasion, EAM) and those of the white dots were measured using complementary software (Artec Scanner, Artec 3D, Luxemburg, European Union) [Fig. 3(a)].

The fiducials, the holes for the probes, and the white dots were also digitized using the FastTrak 3D digitizer [Fig. 3(b)]. The nasion and the EAM were chosen as fiducials because, according to our research experience, they can be identified relatively easily on an anatomical scan without the use of skin markers.

#### 2.3.2 Optical data

Optical data were recorded with a frequency-domain (FD)-NIRS system (Imagent; ISS Inc., Champaign, IL). Data were collected from 15 detectors and 16 source locations. Laser diodes delivered light, modulated at 110 MHz, for each source-location at 690 and 830 nm wavelength (max power: 10 mW, mean power: 1 mW). Out of the 240 combinations, long-distance channels were excluded, resulting in 120 channels with two wavelengths (690 and 830 nm) each. The different diodes were switched on in a time-multiplexing manner. The light from the diodes was transmitted to the scalp using thin optical fibers (diameter=400  μm), whereas the back-scattered light from the head was collected using detector fiber bundles (diameter = 3 mm) connected to photomultiplier tubes (PMTs). Based on heterodyne detection, signal digitization, and fast Fourier Transform, the system generated temporal modulations in the emitted light DC (average), AC (amplitude), and phase. Optical data from all channels were sampled at 39.0625 Hz.

Coupling efficiency between the optodes and the skin, as well as signal noise, were monitored before the beginning of each scan using a complementary software (BOXY, ISS Inc., Champaign, IL) and adjustments were made to maximize the signal quality.

##### Valsalva manoeuver

**Fig. 4:**
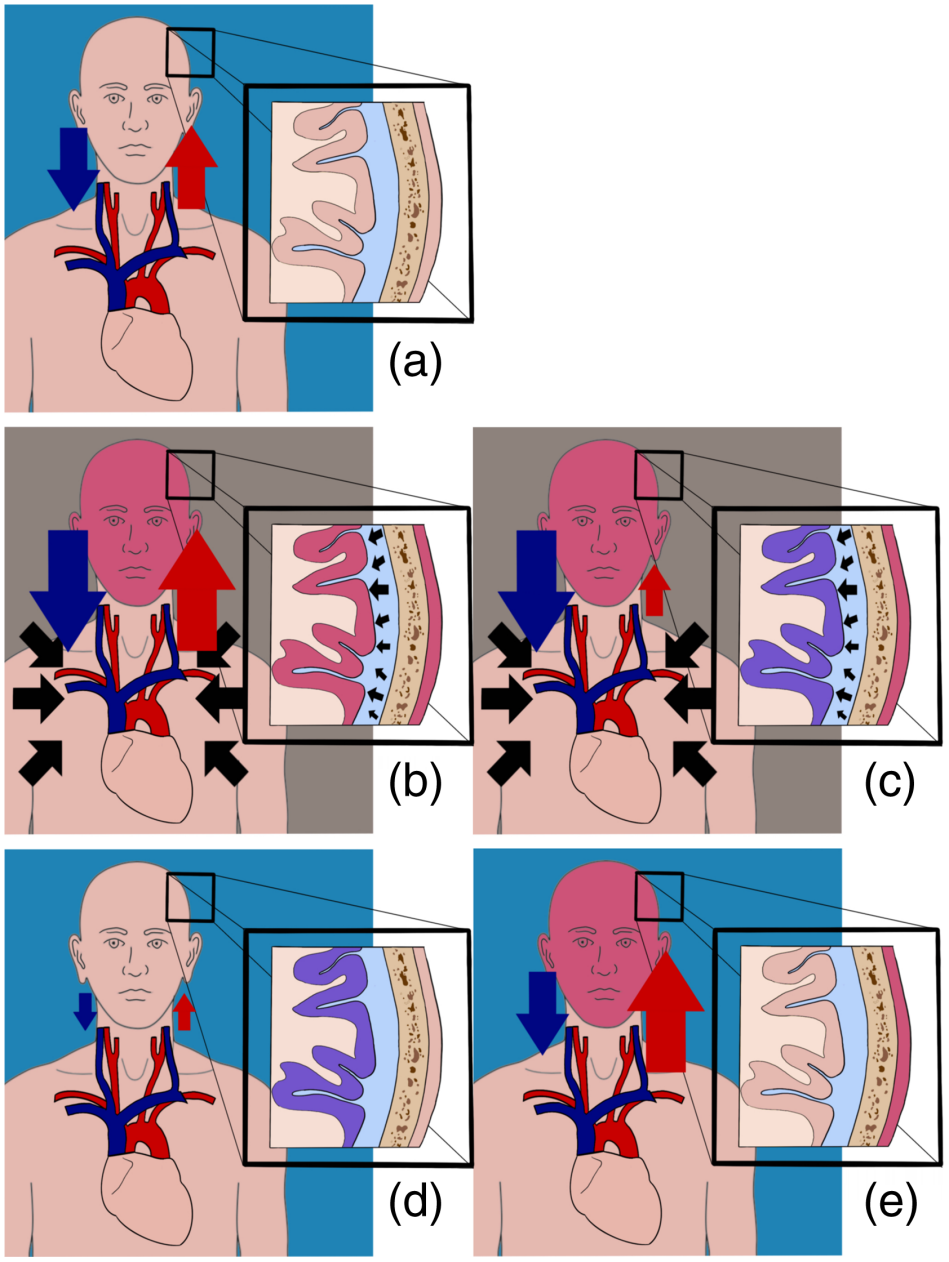
Representation of the physiological changes during the VM phases. (a) Standard physiological state. (b) Phase I. (c) Phase II. (d) Phase III. (e) Phase IV. The blue and the red arrows represent the changes in venous and arterial pressures, respectively. The black arrows in the thorax and those in the cerebrospinal fluid represent the increased intrathoracic and intracranial pressures, respectively, generated during the breath-holding phases of the VM [(b) and (c)]. The blue [(a), (d), and (e)] and gray [(b) and (c)] backgrounds represent an open and closed glottis, respectively. Higher and lower tissue saturations are shown in red and purple, respectively. A detailed description of the VM phases is in the text.

The VM is a forced attempt to exhale against a closed glottis. The detailed knowledge of the expected hemodynamic changes during VM was used to validate the system presented and so is described hereafter. Based on the changes in blood pressure, the VM can be divided into four phases (Fig. 4).^29^

During phase I, lasting ∼2 to 3 s, an increase of intrathoracic pressure, due to the forced exhalation attempt against the closed glottis, causes an increase of external pressure on the main vessels (e.g., aorta, superior vena cava) with consequent increase of the arterial and venous pressures [Fig. 4(b)].^29^^–^^31^ The increase of blood volume (predominantly in the capillaries and veins) causes an increase in the levels of total hemoglobin (HbT) and tissue saturation in the ICT and ECT [Fig. 4(b)].^32^^,^^33^ Due to the confinement of the brain into a nonexpandable space, the increase of cerebral blood volume and the incapacity for it to drain because of the high intrathoracic pressure is immediately followed by a sharp rise in ICP.^34^^–^^36^ The high pressure exerted on the spinal compartment by the thorax is another contributing factor in the increase of ICP.^36^ This simultaneous rise in ICP serves as a protection mechanism for the brain and its blood vessels, which could otherwise be violently damaged by the sudden increase of arterial pressure.^35^^,^^36^ During phase II, the reduction of end-diastolic volume, because of the reduction in venous return due to the high central venous pressure, leads to a decrease of arterial pressure and an increase of heart rate (HR) as a compensatory mechanism [Fig. 4(c)].^29^

The reduction of blood supply, combined with the high brain metabolism, causes a progressive, noncompensated consumption of tissue $O_{2}\mathrm{Hb}$ and a consequent gradual decrease of ICT saturation [Fig. 4(c)].^32^^,^^37^^,^^38^ On the contrary, a similar reduction of tissue saturation is not present in the ECT due to its low level of metabolism [Fig. 4(c)].^38^ During phase III, the glottis is opened, and this is followed by a drop of intrathoracic pressure. The sudden lack of external pressure on the main vessels determines a high level of venous return and a rapid decrease of arterial blood pressure and ICP [Fig. 4(d)].^29^^,^^39^ It is worth noting that, at this stage, the level of arterial blood pressure is at its lowest point.^39^ The large pool of Hb is removed from the ECT by the venous return [Fig. 4(d)]. In ICT, the already low tissue saturation further decreases due to the reduced arterial blood pressure [Fig. 4(d)].^32^ In phase IV, there is a peak of arterial blood pressure and a subsequent increase of the $O_{2}\mathrm{Hb}$ levels in the ECT and ICT [Fig. 4(e)].^29^^,^^32^

The VM was considered a valid task to test the neuromonitoring assessment with fNIRS-DOT, due to the substantial and opposite changes of tissue saturation in ICT and ECT. These two different behaviors may give insight into the ability of the developed DOT approach to uncouple the contributions from the ICT and ECT and therefore test the capacity of the optical technique to record the brain signal without interference from the ECT.^40^^,^^41^ Furthermore, the VM simulates, on healthy human subjects, a common post-brain-trauma scenario, characterized by a decrease of brain saturation and an increase of ICP.^38^^,^^42^^–^^45^

Participants were instructed to perform five VMs, holding their breath for 10 s each time, preceded and followed by 3 min of rest. Ten seconds before each VM, participants were instructed to take as many long gasps as they deemed necessary to prepare for the upcoming physical task. This choice was permitted to mitigate the variability in the task performance between participants based on their level of fitness and commitment to the task.

Participants were in a semi-supine position with their heads slightly tilted by a cushion and in an illuminated environment. We limited the testing to this position as this system is designed for TBI patients with a tier zero or greater management of ICP (i.e., head of bed inclined at 30 deg to 45 deg).^25^ A clinician was observing the participants to reduce the effects of adverse events (e.g., syncope) and to ensure that the VMs were performed correctly. The VMs were monitored by measuring the expected HR increase using a pulse-oximeter, Fingertip (ChoiceMMed, Yuquan Road, Haidian District, Beijing, People’s Republic of China). Participants could stop the experiment at any stage.

### 2.4 Data Analysis

#### 2.4.1 Digitization

The white dots from Artec Leo were co-registered on a structural image template through fiducial alignment based on least square fitting. The optode locations were co-registered by combining the locations of the white dots and the fiducials according to Artec Leo, with the locations of the white dots and the optodes according to the FastTrak 3D digitizer. This method was necessary to mitigate against the potential for inaccurate optode localization and, in extreme cases, optode omission by a direct analysis of the optodes’ and fiducials’ coordinates by Artec Leo. In particular, the measurement of white dots rather than optodes offers the following advantages: (i) the small white dots can be more accurately pinpointed on the Artec Leo images than the large probe housings (especially for detectors), (ii) the greater spread and higher number of white dots than probe housings make it easier to compensate for some missing coordinates due to blurred images, (iii) the use of white dots across the whole head allows one to implement surface-fitting algorithms to minimize the coregistration error even if the probes are potentially tightly clustered together,^18^^,^^19^ and (iv) the subsequent addition of the optodes’ coordinates from an electromagnetic digitization make it possible to account for the probes’ depth from the scanned surface.

The root mean square error (RMSE) between the white dots positions using the Artec Leo image and the FastTrak 3D digitizer was investigated. The number of white dots necessary to optimize the RMSE was calculated.

Finally, the coregistration of the optodes on a template was used for the optical analysis.

#### 2.4.2 Optical data

The AC signal compared with DC has the advantage of being less contaminated by ambient light but carries a lower signal-to-noise ratio. Given that the helmet itself largely shielded the probes from ambient light, the analysis of the DC signal alone was deemed sufficient to address this study’s aims.

The optical DC intensity data were transformed into optical density (OD), movement corrected, and band-pass filtered between 0.01 and 0.5 Hz (zero-lag, 2nd order, Butterworth digital filter).^46^^,^^47^ For each wavelength and channel, in addition to the 10 s of the VM trial, single-trial OD was evaluated from 20 s prior up to 20 s after the VM onset. OD signal, of 50 s duration, was randomly chosen during each rest period to obtain a control measurement. Average OD response for each wavelength, channel, and subject was computed.

For each wavelength, only channels with an OD signal-to-noise ratio (SNR) above 20 were deemed as usable. On average, 78±10 and 72±11 channels for the 830-nm wavelength and the 690-nm wavelength, respectively, were used for further analysis.

Furthermore, a model of light propagation within head (forward model) and an inverse procedure were performed using a structural MRI (MPRAGE sequence) as a template. The FEM applied to the diffusion equation was used to estimate the forward model.^48^^,^^49^ The FEM software NIRFAST was used to model light propagation through the head and to compute Jacobian (sensitivity) matrices of DC light intensity to absorption changes induced by Hb oscillations.^24^^,^^50^ Figure 2(b) reports an example of an average Jacobian (average optical sensitivity) for a participant overlaid onto the anatomical MRI template. The average Jacobian is displayed up to an attenuation of 60 dB (1000 times) from its maximum value. “Fine” meshes (maximum tetrahedral volume $2\;{\mathrm{mm}}^{3}$) were generated for FEM using the MATLAB software iso2mesh.^51^ The heterogeneous head models were based on the segmentation of the anatomical MRI. Segmentation of the skull and scalp, CSF, white matter, and gray matter was performed using statistical parametric mapping (SPM) functions applied to the image.^52^^,^^53^ Baseline optical properties [absorption coefficient ($\mu_{\alpha }$), reduced scattering coefficient ($\mu_{s}$), and refractive index (η)] of the tissues at the relevant wavelength were taken from Tian and Liu.^54^ The Jacobian was computed by interpolation of the “fine” mesh on a “coarse” mesh were each node represented a voxel of $5\times 5\times 5\;{\mathrm{mm}}^{3}$. A linear inverse procedure based on energy minimization of the solution was used to convert intensity changes on individual channels to absorption changes in voxel space at each wavelength.^50^ The Lambert-Beer Law was inverted to evaluate $O_{2}\mathrm{Hb}$ and HHb oscillations given absorption modulation at the two wavelengths employed. The extinction coefficients of the two forms of Hb at the 690 and 830 nm wavelengths were extracted from Zijlstra et al.^55^ Using the computed MRI segmentation, the MRI was separated into ICT and ECT compartments. The ICT compartment was evaluated by summing a smoothed version of white and gray matter segmentation. The ECT compartment was obtained by subtracting a binary image of the head with the previously defined ICT compartment. $O_{2}\mathrm{Hb}$ and HHb signals were evaluated for each subject by averaging the Hb oscillations obtained in the different compartments, considering voxels where enough light sensitivity was obtained [−60 dB of attenuation, Fig. 2(a)].

The general trend in the $O_{2}\mathrm{Hb}$, HHb, and HbT (HbT = $O_{2}\mathrm{Hb}$ + HHb) response across all subjects during the whole VM was qualitatively evaluated. This response could be subject to the different timing and strength of the hemodynamic changes between phases across participants, which cannot be tracked without a continuous arterial pressure monitor. The 10-s window of breath-holding (BH) (i.e., phases I and II) was highlighted. This made it possible to isolate the first two phases, leading to a more effective mapping of the optical data throughout the whole VM.

Because the hemodynamic response during the BH reaches a plateau corresponding to part of phase II, the statistical analysis was limited to the peak of the optical data during the BH so that this could be related with relative certainty to phase II alone for all participants, despite a possible variability in transition timing between phases I and II and the time to reach plateau. Furthermore, as discussed in section “Valsalva manoeuver,” during phase II the changes in tissue saturation between ICT and ECT are opposite, and the brain status simulates a common TBI scenario (i.e., a decrease in brain tissue saturation and an increase in ICP).

A paired t test was performed to assess the effect of the VM on HR. A repeated-measures analysis of variance (rmANOVA) was conducted on hemoglobin changes considering two within-subject factors: condition (VM and rest) and compartment (ICT and ECT). A *post hoc* analysis was performed using paired t tests with false discovery rate (FDR) for multiple comparison correction.

## 3 Results

### 3.1 Coregistration

**Fig. 5:**
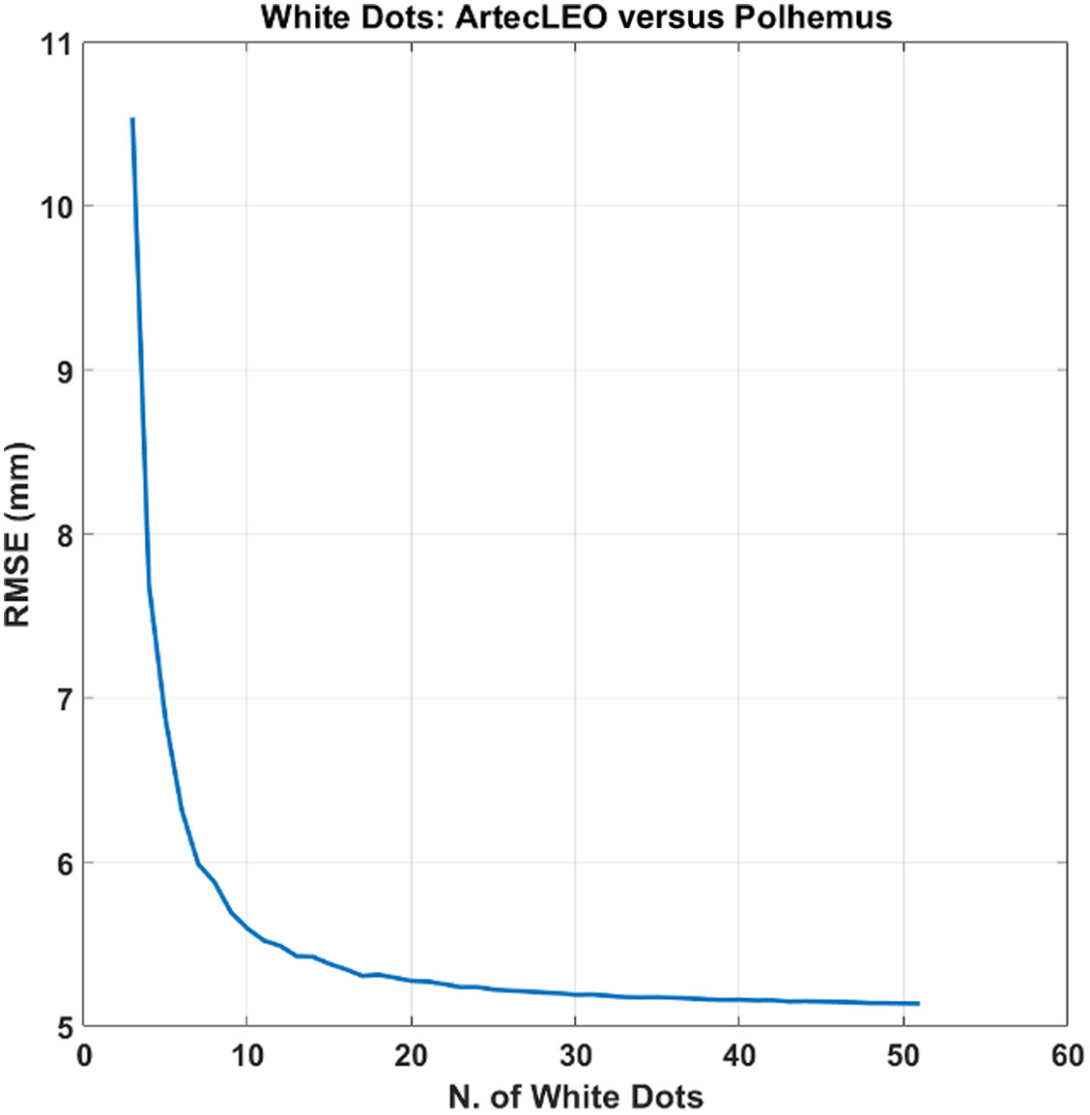
RMSE between Artec Leo and FastTrak for the available white dots (51 in total), as a function of the number of white dots used for coregistration between the two modalities. The RMSE was computed using 10,000 iterations for each number of white dots selected, where the white dots used where randomly chosen.

Figure 5 reports the RMSE between Artec Leo and FastTrak for the available white dots (51 in total), as a function of the number of white dots used for coregistration between the two modalities.

The RMSE reported in the figure was computed using 10,000 iterations for each number of white dots selected, where the white dots used for coregistration in each iteration were randomly chosen from all those available. The minimum number selected was 3. The plot in Fig. 5 clearly shows a plateau in the error between the two modalities with a number of white dots above 10. With this number of dots, the average distance between the corresponding locations is within 5.5 mm.

### 3.2 Optical Data

Five VMs were performed per participant, resulting in a mean increase in HR of 17 bpm (±11) (t=5.17, df=21, $p=3.9\cdot 10^{-5}$).

**Fig. 6:**
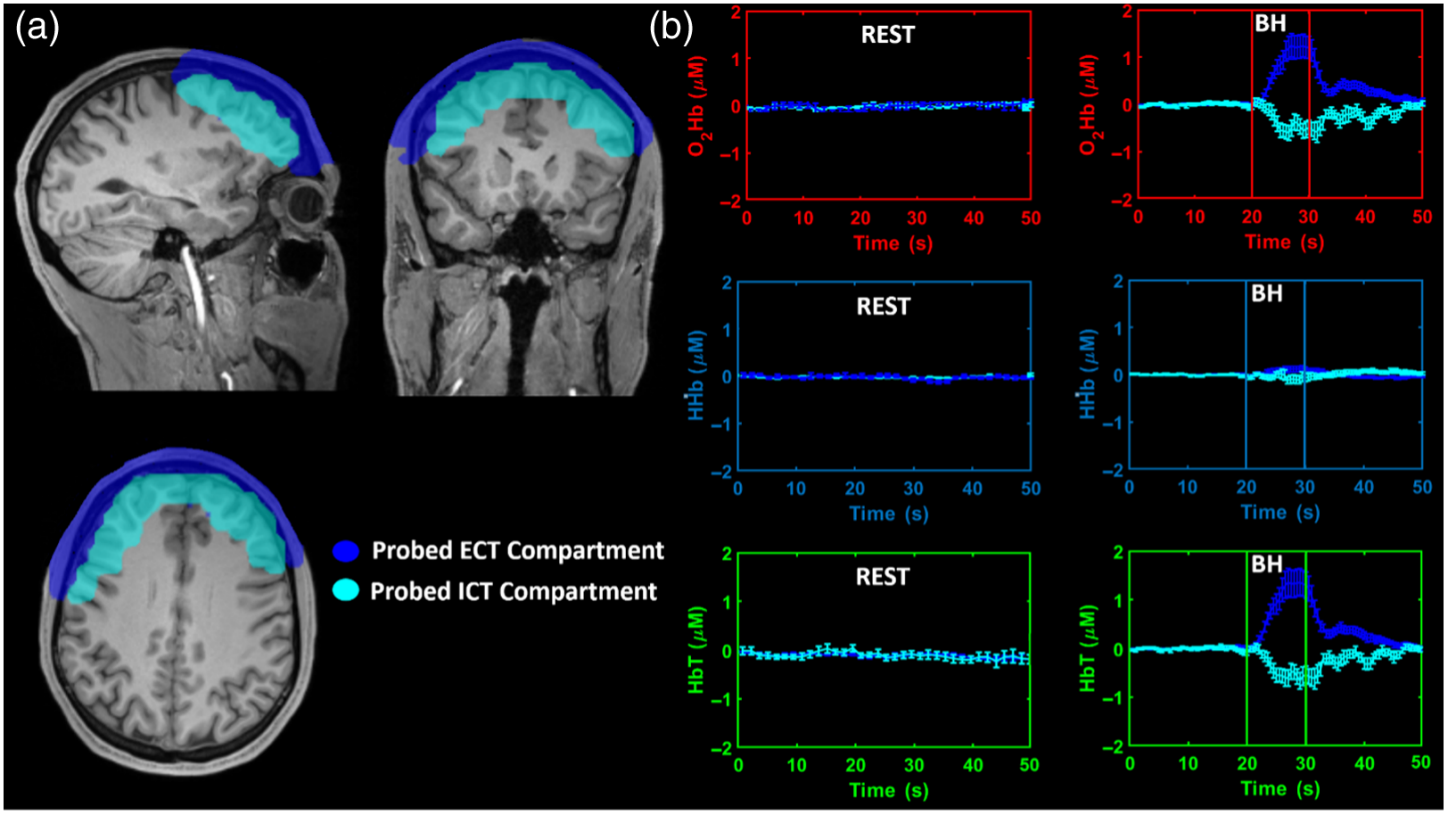
(a) Computed ICT and ECT compartments that were probed by the optical array overlaid on the structural image. (b) Across subjects’ average modulations, and associated standard error, of $O_{2}\mathrm{Hb}$, HHb, and HbT during the rest period and the VM in the two compartments. BH, breath-holding (i.e., phases I and II).

Figure 6(a) reports the ICT and ECT compartments that were probed by the optical array, overlaid onto the structural template image.

The compartments were computed based on the structural MRI employing the algorithm described. Figure 6(b) reports the across subjects’ average modulations, and associated standard error, of $O_{2}\mathrm{Hb}$, HHb, and HbT during the rest period and the VM in the two compartments.

A clear difference in fluctuations of levels of Hb during rest and the VM is visible. Moreover, during the VM, decoupling in the Hb responses between the ICT and ECT compartments is evident. No differences were observed when the ICT and ECT compartments were further separated by right and left hemisphere.

In both these two layers, the changes of $O_{2}\mathrm{Hb}$ and HbT are in agreement with the expected changes of tissue saturation during different VM phases. In the first few seconds of the task, the levels of $O_{2}\mathrm{Hb}$ and HbT began to increase in the ECT and decrease in the ICT. Subsequently, the levels of $O_{2}\mathrm{Hb}$ and HbT in the ECT remained high and low in the ICT. At the time of the breath release (i.e., phase III), the levels of $O_{2}\mathrm{Hb}$ and HbT sharply decreased in the ECT. A reduction in the ICT was less visible, probably because brain saturation was already compromised. In the following seconds (i.e., phase IV), there was a sharp increase of $O_{2}\mathrm{Hb}$ and HbT in the ECT and ICT, with a slight time-delay between the two. Having completed the VM, the parameters of $O_{2}\mathrm{Hb}$ and HbT in the ECT and ICT gradually returned to the values of the resting state.

To test the statistical significance of the results, the peak changes in $O_{2}\mathrm{Hb}$, HHb, and HbT during the BH phases of the VM were extracted. Statistical results are summarized in Table 1.

**Table 1:** Statistical results of the $O_{2}\mathrm{Hb}$, HHb, and HbT changes during the rest period and the BH of the VM, in the ICT, and ECT.

rmANOVA	$O_{2}\mathrm{Hb}$	HHb	HbT
Compartment: ICT versus ECT	F(1,21)=35.51	F(1,21)=0.83	F(1,21)=28.19
	p=∼0	p=n.s.	p=˜0
Condition: VM versus rest	F(1,21)=6.04	F(1,21)=0.53	F(1,21)=7.28
	p=0.023	p=n.s.	p=0.014
Compartment × condition	F(1,21)=35.56	F(1,21)=0.83	F(1,21)=28.42
	p=∼0	p=n.s.	p=∼0
*Post hoc* analysis (paired t test)			
ICT: VM versus rest	t(21)=−3.85		t(21)=−3.74
	$p=9.2\cdot 10^{-4}$		$p={1\cdot 10}^{-3}$
ECT: VM versus rest	t(21)=5.10		t(21)=4.78
	$p=4.7\cdot 10^{-5}$		$p=9.9\cdot 10^{-4}$
VM: ICT versus ECT	t(21)=−5.96		t(21)=−5.32
	$p=6.4\cdot 10^{-6}$		$p=2.8\cdot 10^{-5}$

The rmANOVA showed a significant effect when considering $O_{2}\mathrm{Hb}$ for VM versus rest (F=6.04, df=1−21, p=0.023) and ICT versus ECT (F=35.51, df=1−21, p=˜0), and when considering HbT for VM versus rest (F=7.28, df=1−21, p=0.014) and ICT versus ECT (F=28.19, df=1−21, p=∼0). Importantly, a highly significant interaction between factors was obtained, indicating a synergic effect between condition and compartment for $O_{2}\mathrm{Hb}$ (F=35.56, df=1−21, p=∼0) and HbT (F=28.42, df=1−21, p=∼0). When performing the *post hoc* analysis, a significant decrease in $O_{2}\mathrm{Hb}$ and HbT was observed in the ICT when comparing VM to rest ($O_{2}\mathrm{Hb}$: t=−3.85, df=21, $p=9.2\cdot 10^{-4}$; HbT: t=−3.74, df=21, $p=1.0\cdot 10^{-3}$), whereas a significant increase in $O_{2}\mathrm{Hb}$ and HbT was found in the ECT ($O_{2}\mathrm{Hb}$: t=5.10, df=21, $p=4.7\cdot 10^{-5}$; HbT: t=4.78, df=21, $p=9.9\cdot 10^{-4}$).

Moreover, when comparing the ECT and the ICT during the VM, a significant difference was obtained for both $O_{2}\mathrm{Hb}$ (t=5.96, df=21, $p=6.4\cdot 10^{-6}$) and HbT (t=5.32, df=21, $p=2.8\cdot 10^{-5}$).

## 4 Discussion

### 4.1 Coregistration

The developed approach yielded an average displacement error, expressed as RMSE, of 5.5 mm (Fig. 5), which importantly is well below the spatial resolution limit of DOT of ∼15  mm.^14^ This means that the Artec Leo images, through the method presented here, can be applied to obtain an accurate coregistration of the optodes onto a subject-specific anatomical image or an anatomical template, with similar performance to an electromagnetic digitization. The maximum benefit of accurate coregistration to anatomical images would occur when subject-specific images are available. Nonetheless, reliable results at a group level can also be obtained by an accurate coregistration to a template. The degree of error between the two techniques may differ slightly from the 5.5-mm RMSE measured. The coordinates from Artec Leo can fail to exactly match the digitized spots within the area of the white dots, so the absolute value of RMSE could be partially due to the testing analysis itself rather than the method implemented. However, this miscalculation can also affect the optical analysis, as the optode coregistration by Artec Leo is based on the measured distances between white dots and optodes by the electromagnetic digitization. The small sizes of the white dots mitigate, to a greater or less extent, this error. The housings’ width, especially for the detectors, can be another cause of inaccuracy in the optical analysis, because there could be a mismatch between the digitized spot within the housings and the real locus of the optodes. The measurement of the digitization error was limited to the coregistration accuracy, without considering the accuracy in mapping the hemodynamic signal itself. A probe displacement error would likely increase the aforementioned spatial resolution limit of DOT. However, this increase would be marginal compared to the intrinsic limitation of the optical technique itself.^14^

Remarkably, this method can be performed without additional CT scans beyond the one performed upon admission for clinical assessment. The coregistration to the initial CT scan allows clinicians to prioritize the optical analysis of the most affected and clinically significant regions, as well as neuromonitoring from the early stages of injury, when secondary injuries are more prone to happen.^33^ It should be mentioned that the ability to assess the optical properties of a brain lesion would depend on its size, so lesions smaller than the margin of error between the two coregistrations are likely to be incorrectly located or overlooked; however, this problem is inherent to the limited spatial resolution of DOT.^14^ The decoupling of the commencement of DOT recordings from acquiring a dedicated anatomical image, as well as being useful for routine TBI patient care, could also prove paramount in extreme cases: in the most severe cases of TBI patients whose ICP would be difficult to control in the scanner or with extracorporeal systems (e.g., hemodialysis), and for whom repetitive CT scans are thus inaccessible, or alternatively in cases where clinicians are prioritizing other patients and a second anatomical imaging has to be postponed.

It is important to stress that the method reported here relies upon measured distances between the white dots and the optodes from a previously performed electromagnetic digitization. Hence, the developed approach for the coregistration of optical data on TBI patients in the ICU would require digitizing the white dots and the optical array on a phantom head or a healthy volunteer, with a head circumference similar to that of the subject that is going to be measured. Using a digitized helmet, the measurements performed on the TBI patient using an Artec Leo image would allow clinicians to correctly locate the array onto the patient’s CT scan or MRI, where the same fiducials can be selected.

The analysis was performed on white dots that were uniformly distributed around the optode locations. Thus, it is essential for future helmets to maintain the uniformity of white dots in their design to accurately recover the optode locations on the anatomical image. Although the results show a plateau of RMSE using 10 or more white dots, applications of this method on TBI for research purposes suggest that helmets should possess as many white dots as can be fitted onto the exposed surface. This is because acquiring an optimal 3D image may not always be possible in hospital settings, and blurred images may impair the retrieval of all the white dots, with detrimental consequences on the minimum number (i.e., 10) of white dots and the uniformity of their distribution across the head. Moreover, the availability of white dots uniformly covering not only the area around the optodes (as presented herein), but the whole head surface, may allow one to employ more complex algorithms that may further reduce the error in optode positioning.^18^^,^^19^

### 4.2 Optical Data

The fluctuations of $O_{2}\mathrm{Hb}$, HHb, and HbT levels in the ICT due to the VM were separated in the analysis from those in the ECT. In both layers, the results in $O_{2}\mathrm{Hb}$ and HbT conformed with the expected changes during the different phases of the VM. This consistency validates the optical system and the proposed analytical framework as being capable of monitoring changes in brain optical properties due to Hb oscillations (fNIRS-DOT). The rather stable HHb in both layers can be to the well-known poorer signal-to-noise ratio of HHb compared to $O_{2}\mathrm{Hb}$.^56^ Despite fNIRS being unable to quantitatively track saturation, the obtained changes in $O_{2}\mathrm{Hb}$, HHb, and HbT levels are compatible with saturation changes of few points percent if standard baseline values are assumed, with negative and positive changes in the ICT and ECT, respectively.

A decrease of cerebral saturation during VM was also reported in studies that used NIRS devices.^32^^,^^38^^,^^40^^,^^57^^–^^59^ However, these analyses were limited to measurement of the decrease of cerebral saturation, and the ECT signal plausibly contaminated the ICT signal by an unknown value. On the contrary, the fNIRS-DOT analysis reported herein allowed for a comparison of the changes of levels of Hb in the ICT and ECT, providing a better understanding of how the detected optical signal relates to brain physiology. Similarly to other studies, we were unable to quantify the role played by the ECT in the ICT signal; however, the strong hemodynamic signal in the ICT leads us to think that no matter the extent of this contamination, it would be insufficient to obscure the tracking of the ICT signal during the VM.^60^ We believe that our test validated the aims of the study: (i) to test the ability of ICT monitoring and (ii) to separate the ICT-ECT signals.

Acute brain trauma is prone to significant injury evolution of pre-existing lesions (e.g., traumatic penumbra, HPC) and new injuries emerging in previously seemingly unaffected brain regions (e.g., ischemic stroke, delayed traumatic intracerebral hemorrage).^33^^,^^61^^–^^65^ Changes in the optical properties can be used to monitor these potential evolutions and aid clinicians to adjust treatments. Ischemic brain lesion assessment which relies on absolute values of saturation can mislead clinicians due to (i) a lack of complete representation of the tissue respiration by optical assessment alone and (ii) high interindividual variability.^8^^,^^11^ Both of these factors make it impossible to establish a universal saturation threshold indicative of the presence of ischemia (as measured by ${\mathrm{PbtO}}_{2}$ monitoring), or an amount of change, which would indicate deterioration of the existing ischemic areas or the emergence of new ones.^8^ The ICT monitoring presented herein would only track changes relative to an initial value, which would itself differ by an unknown amount from the “healthy” starting point in that brain area for that particular TBI patient. The optical analysis is therefore intended to be included in a multimodal approach which comprises (i) functional and structural, (ii) systemic and neuro, (iii) invasive and noninvasive, and (iv) fNIRS-DOT and contrast-enhanced DOT monitoring, to give a comprehensive and accurate interpretation of the changes in optical properties.^11^

The reading of a CT scan by an experienced clinician is a highly effective method to assess and predict the brain status and to adjust treatment accordingly.^66^ A change in saturation in areas known to be prone to evolution (e.g., traumatic penumbra, oedema) because of their structural presentation could be correctly interpreted by clinicians even if the magnitude of this change is smaller than in areas where an initial injury is not present. By contrast, changes in optical properties in regions previously considered unaffected and “healthy,” especially if there are compounding systemic (e.g., coagulopathy) and neurological risk factors (e.g., subarachnoid hemorrage, subdural hematoma, initial size of contusion), could prompt repetitive CT scans to track possible deterioration.^62^^,^^67^^–^^69^ On top of tracking changes in tissue saturation because of ischemia, changes in optical density over time or between contralateral areas can also be used to assess the evolution or new formation of intracranial hemorrage.^27^^,^^70^ Because the illuminated volume can comprise both ischemic and hemorrhagic areas, the assessment of the tissue oxygenation by absolute value of tissue saturation or its relative change may be significantly hampered by the high light absorption of the hemorragic component, which causes changes in illuminated volume and deceptive value of tissue saturation. The analysis of the CT scan and the signal coregistration would give a correct interpretation of the optical signal.

Changes in optical properties can also be useful in time-limited cerebral autoregulation assessment subject to arterial blood pressure challenges or to observe the effects of spontaneous blood pressure oscillation during paroxysimpatic dysregulation and thus adapt the intensity of analgesia accordingly.^71^^,^^72^

The results presented herein are in agreement with a pilot study, which was performed without the extensive use of short channels.^73^ This leads us to believe that the coregistration process implemented in both studies is a critical component in these optical results. This is in agreement with other studies, which showed that co-registering the optical signal with a structural image could improve the imaging resolution and the localization accuracy.^74^^,^^75^ Furthermore, Clancy et al.^76^^,^^77^ specifically reported an improvement in the accuracy of the signal detected during VM in simulated studies, by co-registering the optical data with a structural image. However, in a study on a healthy volunteer, the coregistration of the optical signal from a high-density patch into an atlas did not yield the same capacity to retrieve physiological changes of tissue saturation associated with the VM as in the simulations.^78^ This limited capacity in an *in vivo* analysis may be explained in part or in whole by a suboptimal coregistration process compared with the coregistration system presented herein. Layer thicknesses and optical properties can vary significantly between individuals and across different areas of the head.^79^^,^^80^ These differences can be even more significant in TBI patients due to brain lesions (e.g., ischemia, hemorrhage) or surgical interventions (e.g., decompressive craniectomy).^11^ The coregistration of the optical analysis into a subject-specific image accounts for the individual anatomy of these layers and brain lesions. To sum up, the coregistration of the optical signal with a subject-specific image may be particularly important for an accurate clinical interpretation of the optical parameters, as well as to enhance the optical signal itself.

### 4.3 Limitations

#### 4.3.1 Absence of a continuous arterial blood pressure monitoring

Grading the hemodynamic changes, if indeed there were any, based on the changes in blood pressure was not possible. This may have affected the results as the quality of the performance of the VM may have differed across participants due to varying levels of cardiovascular fitness and commitment to the task. It is also not possible to perform the VM in the supine position at the same intensity as in the orthostatic position.^30^^,^^39^ This furthers the risk of a poor hemodynamic response across participants if the VM is not performed with sufficient strength. As explained in section “Valsalva manoeuver,” this was mitigated by an individualized preparation for the task (e.g., taking long gasps). Furthermore, the HR response showed an overall increase consistent with the expected hemodynamic changes.

As already discussed in Sec. 2.4.2, the lack of a continuous blood pressure monitoring also prevented the separation of the different phases of the VM. However, the distinction between BH and non-BH phases gave valuable insight into the different hemodynamic moments.

#### 4.3.2 Global hemodynamic response during Valsalva manoeuver

The cerebral perfusion and tissue saturation in TBI patients can vary significantly across the brain, whereas the VM triggers a similar hemodynamic response across the whole brain.^81^^,^^82^ As a matter of fact, no differences were detected between hemispheres. Therefore, the ability to separate the different hemodynamic across the brain of TBI patients could not be determined from the results obtained. As discussed in Sec. 4.1, this capability would likely depend on the spatial resolution limit of the type of DOT applied (e.g., number of channels) far more than the application of the coregistration method presented herein.

#### 4.3.3 Absence of subject-specific structural image

An MRI template was used in place of the subject-specific structural image for the coregistration of the optical data. This reduced the accuracy of the fitting procedure of the probes on the participants’ scalps due to their different head shapes. Moreover, the approximation also affected the optical data analysis because the assumed optical properties of the different head layers were probably not anatomically identical among subjects (e.g., layer thickness).

As discussed in Sec. 4.2, the coregistration could have enhanced the optical results. This could be even more advantageous if a subject-specific structural image were used, especially with a distorted structural and functional anatomy, as in TBI.

#### 4.3.4 Limited field of view

Although studies which investigated a broader coverage of the head using DOT have been presented for clinical applications in non-ICU scenarios (e.g., Parkinson syndrome), we limited the analysis to the frontal lobes to address the standard of care in ICU.^50^ In the future, new studies with a broader field of acquisition should be implemented while maintaining the standard of care in acute TBI patients.

#### 4.3.5 Limited number of probes and wavelengths

Our study was limited to 15 detectors and 16 sources at two wavelengths (690 and 830 nm) mainly designed to measure $O_{2}\mathrm{Hb}$ and HHb levels. Future studies should apply large field-of-view HD (high density) DOT to further improve the optical accuracy, as well as using different wavelengths to make the recording more suitable for ICG measurement.^6^

#### 4.3.6 Absence of phase-shift analysis

Although a frequency-domain device was used, neither AC nor phase data were implemented into the optical analysis. From results on simulated and phantom studies, analysis of the phase-shift can, theoretically, further increase imaging resolution and accuracy.^83^^,^^84^ Future studies should explore this potentiality.

## 5 Conclusion

The results suggest that our method is capable of addressing the foreseeable problems associated with DOT neuromonitoring in TBI patients in the ICU. This procedure could enable fNIRS-DOT and contrast-enhanced DOT recordings as part of a multimodal monitoring on TBI patients upon hospital admission.

Future research should test other probe-position geometries, including large field-of-view HD-DOT, which may further increase the capability of the optical analysis to separate the signal from the different head layers and to monitor brain lesions. Clinical studies should be designed so that DOT measurements are included in a multimodal monitoring.

## Data Availability

Code and anonymized optical data are available in MATLAB format in FigShare at https://doi.org/10.6084/m9.figshare.25866682.v1.

## References

[1] Maas A. I. R. et al., “Traumatic brain injury: integrated approaches to improve prevention, clinical care, and research,” Lancet Neurol. 16(12), 987–1048 (2017). 10.1016/S1474-4422(17)30371-X 29122524

[2] Davies D. J. et al., “Near-infrared spectroscopy in the monitoring of adult traumatic brain injury: a review,” J. Neurotrauma 32(13), 933–941 (2015). JNEUE4 0897-7151 10.1089/neu.2014.3748 25603012 PMC4492772

[r3] Green M. S. Sehgal S. Tariq R., “Near-infrared spectroscopy: the new must have tool in the intensive care unit?” Semin. Cardiothorac. Vasc. Anesth. 20(3), 213–224 (2016). 10.1177/1089253216644346 27206637

[4] Haitsma I. K. Maas A. I., “Monitoring cerebral oxygenation in traumatic brain injury,” Prog. Brain Res. 161, 207–216 (2007). PBRRA4 0079-6123 10.1016/S0079-6123(06)61014-5 17618979

[5] Forcione M. et al., “Cerebral perfusion and blood–brain barrier assessment in brain trauma using contrast-enhanced near-infrared spectroscopy with indocyanine green: a review,” J. Cereb. Blood Flow Metab. 40(8), 1586–1598 (2020). 10.1177/0271678X20921973 32345103 PMC7370372

[r6] Forcione M. et al., “Dynamic contrast-enhanced near-infrared spectroscopy using indocyanine green on moderate and severe traumatic brain injury: a prospective observational study,” Quant. Imaging Med. Surg. 10(11), 2085–2097 (2020). 10.21037/qims-20-742 33139989 PMC7547258

[7] Ganau M. et al., “Breakthrough in the assessment of cerebral perfusion and vascular permeability after brain trauma through the adoption of dynamic indocyanin green-enhanced near-infrared spectroscopy,” Quant. Imaging Med. Surg. 10(11), 2081–2084 (2020). 10.21037/qims-20-905 33141119 PMC7547265

[8] Davies D. J. et al., “Cerebral oxygenation in traumatic brain injury: can a non-invasive frequency domain near-infrared spectroscopy device detect changes in brain tissue oxygen tension as well as the established invasive monitor?” J. Neurotrauma 36(7), 1175–1183 (2019). JNEUE4 0897-7151 10.1089/neu.2018.5667 29877139

[r9] Leal-Noval S. R. et al., “Invasive and noninvasive assessment of cerebral oxygenation in patients with severe traumatic brain injury,” Intensive Care Med. 36(8), 1309–1317 (2010). ICMED9 0342-4642 10.1007/s00134-010-1920-7 20502869

[10] Weigl W. et al., “Application of optical methods in the monitoring of traumatic brain injury: a review,” J. Cereb. Blood Flow Metab. 36(11), 1825–1843 (2016). 10.1177/0271678X16667953 27604312 PMC5094301

[11] Forcione M. et al., “Mismatch between tissue partial oxygen pressure and near-infrared spectroscopy neuromonitoring of tissue respiration in acute brain trauma: the rationale for implementing a multimodal monitoring strategy,” Int. J. Mol. Sci. 22(3), 1122 (2021).1422-0067 10.3390/ijms22031122 33498736 PMC7865258

[12] Bigio I. J. Fantini S., Quantitative Biomedical Optics: Theory, Methods, and Applications, Cambridge University Press, Cambridge (2016).

[r13] Wheelock M. D. Culver J. P. Eggebrecht A. T., “High-density diffuse optical tomography for imaging human brain function,” Rev. Sci. Instrum. 90(5), 051101 (2019). RSINAK 0034-6748 10.1063/1.5086809 31153254 PMC6533110

[14] Chiarelli A. M. et al., “Combining energy and Laplacian regularization to accurately retrieve the depth of brain activity of diffuse optical tomographic data,” J. Biomed. Opt. 21(3), 036008 (2016). JBOPFO 1083-3668 10.1117/1.JBO.21.3.036008 26987429 PMC4796096

[15] Austin T. et al., “Three dimensional optical imaging of blood volume and oxygenation in the neonatal brain,” NeuroImage 31(4), 1426–1433 (2006). NEIMEF 1053-8119 10.1016/j.neuroimage.2006.02.038 16644237

[16] Chiarelli A. M. et al., “Assessment of cerebrovascular development and intraventricular hemorrhages in preterm infants with optical measures of the brain arterial pulse wave,” J. Cereb. Blood Flow Metab. 39(3), 466–480 (2019). 10.1177/0271678X17732694 28949275 PMC6421243

[17] Giacalone G. et al., “Time-domain near-infrared spectroscopy in acute ischemic stroke patients,” Neurophotonics 6(1), 015003 (2019). 10.1117/1.NPh.6.1.015003 30796883 PMC6365799

[r18] Whalen C. et al., “Validation of a method for coregistering scalp recording locations with 3D structural MR images,” Hum. Brain Mapp. 29(11), 1288–1301 (2008). HBRME7 1065-9471 10.1002/hbm.20465 17894391 PMC6871211

[19] Chiarelli A. M. et al., “Comparison of procedures for co-registering scalp-recording locations to anatomical magnetic resonance images,” J. Biomed. Opt. 20(1), 016009 (2015). JBOPFO 1083-3668 10.1117/1.JBO.20.1.016009 25574993 PMC4288136

[20] Birkfellner W. et al., “Systematic distortions in magnetic position digitizers,” Med. Phys. 25(11), 2242–2248 (1998). MPHYA6 0094-2405 10.1118/1.598425 9829253

[21] Moore E. E. Feliciano D. V. Mattox K. L., Trauma, 8th ed., McGraw-Hill Education (2017).

[22] Tan C. H. et al., “Mapping cerebral pulse pressure and arterial compliance over the adult lifespan with optical imaging,” PLoS One 12(2), e0171305 (2017). POLNCL 1932-6203 10.1371/journal.pone.0171305 28234912 PMC5325189

[23] He L. et al., “Noninvasive continuous optical monitoring of absolute cerebral blood flow in critically ill adults,” Neurophotonics 5(4), 045006 (2018). 10.1117/1.NPh.5.4.045006 30480039 PMC6251207

[24] Dehghani H. et al., “Near infrared optical tomography using NIRFAST: algorithm for numerical model and image reconstruction,” Commun. Numer. Methods Eng. 25(6), 711–732 (2008). CANMER 0748-8025 10.1002/cnm.1162 20182646 PMC2826796

[25] Hawryluk G. W. J. et al., “A management algorithm for patients with intracranial pressure monitoring: the Seattle international severe traumatic brain injury consensus conference (SIBICC),” Intensive Care Med. 45(12), 1783–1794 (2019). ICMED9 0342-4642 10.1007/s00134-019-05805-9 31659383 PMC6863785

[26] Mathewson K. E. et al., “Dynamics of alpha control: preparatory suppression of posterior alpha oscillations by frontal modulators revealed with combined EEG and event-related optical signal,” J. Cogn. Neurosci. 26(10), 2400–2415 (2014). JCONEO 0898-929X 10.1162/jocn_a_00637 24702458 PMC4291167

[27] Robertson C. S. et al., “Clinical evaluation of a portable near-infrared device for detection of traumatic intracranial hematomas,” J. Neurotrauma 27(9), 1597–1604 (2010). JNEUE4 0897-7151 10.1089/neu.2010.1340 20568959

[28] Forcione M. et al., “Tomographic task-related functional near-infrared spectroscopy in acute sport-related concussion: an observational case study,” Int. J. Mol. Sci. 21(17), 6273 (2020).1422-0067 10.3390/ijms21176273 32872557 PMC7503954

[29] Tiecks F. P. et al., “Effects of the Valsalva maneuver on cerebral circulation in healthy adults: a transcranial Doppler study,” Stroke 26(8), 1386–1392 (1995). SJCCA7 0039-2499 10.1161/01.STR.26.8.1386 7631342

[r30] Pott F. et al., “Middle cerebral artery blood velocity during a Valsalva maneuver in the standing position,” J. Appl. Physiol. 88(5), 1545–1550 (2000). 10.1152/jappl.2000.88.5.1545 10797110

[31] Perry B. G. Lucas S. J. E., “The acute cardiorespiratory and cerebrovascular response to resistance exercise,” Sports Med. Open 7(1), 36 (2021). 10.1186/s40798-021-00314-w 34046740 PMC8160070

[32] Perry B. G. et al., “Cerebral hemodynamics during graded Valsalva maneuvers,” Front. Physiol. 5, 349 (2014). FROPBK 0301-536X 10.3389/fphys.2014.00349 25309449 PMC4163977

[33] Caplan L. R. et al., Primer on Cerebrovascular Diseases, 2nd ed., Academic Press (2017).

[34] Wilson M. H., “Monro-Kellie 2.0: the dynamic vascular and venous pathophysiological components of intracranial pressure,” J. Cereb. Blood Flow Metab. 36(8), 1338–1350 (2016). 10.1177/0271678X16648711 27174995 PMC4971608

[r35] Prabhakar H. et al., “Intracranial pressure changes during Valsalva manoeuvre in patients undergoing a neuroendoscopic procedure,” Minim. Invasive Neurosurg. 50(2), 98–101 (2007). 10.1055/s-2007-982505 17674296

[36] Haykowsky M. J. et al., “Resistance exercise, the Valsalva maneuver, and cerebrovascular transmural pressure,” Med. Sci. Sports Exerc. 35(1), 65–68 (2003). 10.1097/00005768-200301000-00011 12544637

[37] Greenfield J. C. Jr. Rembert J. C. Tindall G. T., “Transient changes in cerebral vascular resistance during the Valsalva maneuver in man,” Stroke 15(1), 76–79 (1984). SJCCA7 0039-2499 10.1161/01.STR.15.1.76 6229907

[38] Davies D. et al., “Comparison of near infrared spectroscopy with functional MRI for detection of physiological changes in the brain independent of superficial tissue,” Lancet 387, S34 (2016). LANCAO 0140-6736 10.1016/S0140-6736(16)00421-9

[39] Perry B. G. et al., “The cerebrovascular response to graded Valsalva maneuvers while standing,” Physiol. Rep. 2(2), e00233 (2014). 10.1002/phy2.233 24744902 PMC3966248

[40] Davies D. J. et al., “The Valsalva maneuver: an indispensable physiological tool to differentiate intra versus extracranial near-infrared signal,” Biomed. Opt. Express 11(4), 1712–1724 (2020). BOEICL 2156-7085 10.1364/BOE.11.001712 32341842 PMC7173884

[41] Steinbrink J. et al., “Determining changes in NIR absorption using a layered model of the human head,” Phys. Med. Biol. 46(3), 879–896 (2001). PHMBA7 0031-9155 10.1088/0031-9155/46/3/320 11277232

[42] Davies D. J., “Cerebral near infra-red spectroscopy in traumatic brain injury as a potential independent monitoring modality and alternative to invasive tissue oxygen tension sensors,” School of Clinical and Experimental Medicine, University of Birmingham UBIRA E THESIS (2017).

[r43] Lee K. J. et al., “Non-invasive detection of intracranial hypertension using a simplified intracranial hemo- and hydro-dynamics model,” Biomed. Eng. Online 14(1), 51 (2015). 10.1186/s12938-015-0051-3 26024843 PMC4449568

[r44] Mousavi S. R. et al., “Measurement of in vivo cerebral volumetric strain induced by the Valsalva maneuver,” J. Biomech. 47(7), 1652–1657 (2014). JBMCB5 0021-9290 10.1016/j.jbiomech.2014.02.038 24656483

[45] Ertl-Wagner B. B. et al., “Demonstration of periventricular brain motion during a Valsalva maneuver: description of technique, evaluation in healthy volunteers and first results in hydrocephalic patients,” Eur. Radiol. 11(10), 1998–2003 (2001). 10.1007/s003300100941 11702134

[46] Chiarelli A. M. et al., “A kurtosis-based wavelet algorithm for motion artifact correction of fNIRS data,” NeuroImage 112, 128–137 (2015). NEIMEF 1053-8119 10.1016/j.neuroimage.2015.02.057 25747916 PMC4408240

[47] Perpetuini D. et al., “A motion artifact correction procedure for fNIRS signals based on wavelet transform and infrared thermography video tracking,” Sensors 21(15), 5117 (2021). SNSRES 0746-9462 10.3390/s21155117 34372353 PMC8346954

[48] Ishimaru A., “Diffusion of light in turbid material,” Appl. Opt. 28(12), 2210–2215 (1989). APOPAI 0003-6935 10.1364/AO.28.002210 20555501

[49] Paulsen K. D. Jiang H., “Spatially varying optical property reconstruction using a finite element diffusion equation approximation,” Med. Phys. 22(6), 691–701 (1995). MPHYA6 0094-2405 10.1118/1.597488 7565358

[50] Eggebrecht A. T. et al., “Mapping distributed brain function and networks with diffuse optical tomography,” Nat. Photonics 8(6), 448–454 (2014). NPAHBY 1749-4885 10.1038/nphoton.2014.107 25083161 PMC4114252

[51] Qianqian F. Boas D. A., “Tetrahedral mesh generation from volumetric binary and grayscale images,” in Proc. IEEE Int. Symp. Biomed. Imaging: From Nano to Macro, pp. 1142–1145 (2009). 10.1109/ISBI.2009.5193259

[52] Friston K. J. et al., “Statistical parametric maps in functional imaging: a general linear approach,” Hum. Brain Mapp. 2(4), 189–210 (1994). HBRME7 1065-9471 10.1002/hbm.460020402

[53] Penny W. et al., Statistical Parametric Mapping: The Analysis of Functional Brain Images, Elsevier, Amsterdam (2011).

[54] Tian F. Liu H., “Depth-compensated diffuse optical tomography enhanced by general linear model analysis and an anatomical atlas of human head,” NeuroImage 85 Pt 1, 166–180 (2014). NEIMEF 1053-8119 10.1016/j.neuroimage.2013.07.016 23859922 PMC4524535

[55] Zijlstra W. G. Buursma A. Meeuwsen-van der Roest W. P., “Absorption spectra of human fetal and adult oxyhemoglobin, de-oxyhemoglobin, carboxyhemoglobin, and methemoglobin,” Clin. Chem. 37(9), 1633–1638 (1991). 10.1093/clinchem/37.9.1633 1716537

[56] Bluestone A. et al., “Three-dimensional optical tomography of hemodynamics in the human head,” Opt. Express 9(6), 272–286 (2001). OPEXFF 1094-4087 10.1364/OE.9.000272 19421298

[57] Clancy M. et al., “Comparison of neurological NIRS signals during standing Valsalva maneuvers, pre and post vasopressor injection,” Proc. SPIE 9538, 953817 (2015). PSISDG 0277-786X 10.1117/12.2183796

[r58] Canova D. et al., “Inconsistent detection of changes in cerebral blood volume by near infrared spectroscopy in standard clinical tests,” J. Appl. Physiol. 110(6), 1646–1655 (2011). 10.1152/japplphysiol.00003.2011 21474700

[59] Saager R. Berger A., “Measurement of layer-like hemodynamic trends in scalp and cortex: implications for physiological baseline suppression in functional near-infrared spectroscopy,” J. Biomed. Opt. 13(3), 034017 (2008). JBOPFO 1083-3668 10.1117/1.2940587 18601562

[60] Eleveld N. et al., “The influence of extracerebral tissue on continuous wave near-infrared spectroscopy in adults: a systematic review of in vivo studies,” J. Clin. Med. 12(8), 2776 (2023). 10.3390/jcm12082776 37109113 PMC10146120

[61] Kurland D. et al., “Hemorrhagic progression of a contusion after traumatic brain injury: a review,” J. Neurotrauma 29(1), 19–31 (2012). JNEUE4 0897-7151 10.1089/neu.2011.2122 21988198 PMC3253310

[r62] Alahmadi H. Vachhrajani S. Cusimano M. D., “The natural history of brain contusion: an analysis of radiological and clinical progression,” J. Neurosurg. 112(5), 1139–1145 (2010). JONSAC 0022-3085 10.3171/2009.5.JNS081369 19575576

[r63] Newcombe V. F. J. et al., “Microstructural basis of contusion expansion in traumatic brain injury: insights from diffusion tensor imaging,” J. Cereb. Blood Flow Metab. 33(6), 855–862 (2013). 10.1038/jcbfm.2013.11 23423189 PMC3677102

[r64] Narayan R. K. et al., “Progression of traumatic intracerebral hemorrhage: a prospective observational study,” J. Neurotrauma 25(6), 629–639 (2008). JNEUE4 0897-7151 10.1089/neu.2007.0385 18491950

[65] Liu S. et al., “Posttraumatic cerebral infarction in severe traumatic brain injury: characteristics, risk factors and potential mechanisms,” Acta Neurochir. 157(10), 1697–1704 (2015). 10.1007/s00701-015-2559-5 26306582

[66] Chesnut R. M. et al., “A trial of intracranial-pressure monitoring in traumatic brain injury,” N. Engl. J. Med. 367(26), 2471–2481 (2012). NEJMAG 0028-4793 10.1056/NEJMoa1207363 23234472 PMC3565432

[67] Fainardi E. et al., “Time course of CT evolution in traumatic subarachnoid haemorrhage: a study of 141 patients,” Acta Neurochir. 146(3), 257–263 (2004). 10.1007/s00701-003-0207-y 15015048

[r68] Chieregato A. et al., “Factors associated with neurological outcome and lesion progression in traumatic subarachnoid hemorrhage patients,” Neurosurgery 56(4), 671–680 (2005). NEQUEB 10.1227/01.NEU.0000156200.76331.7A 15792505

[69] Maegele M. et al., “Coagulopathy and haemorrhagic progression in traumatic brain injury: advances in mechanisms, diagnosis, and management,” Lancet Neurol. 16(8), 630–647 (2017). 10.1016/S1474-4422(17)30197-7 28721927

[70] Robertson C. S. Gopinath S. Chance B., “Use of near infrared spectroscopy to identify traumatic intracranial hemotomas,” J. Biomed. Opt. 2(1), 31–41 (1997). JBOPFO 1083-3668 10.1117/12.261680 23014820

[71] Klein S. P. Depreitere B. Meyfroidt G., “How I monitor cerebral autoregulation,” Crit. Care 23(1), 160 (2019). 10.1186/s13054-019-2454-1 31064383 PMC6505261

[72] Meyfroidt G. Baguley I. J. Menon D. K., “Paroxysmal sympathetic hyperactivity: the storm after acute brain injury,” Lancet Neurol. 16(9), 721–729 (2017). 10.1016/S1474-4422(17)30259-4 28816118

[73] Forcione M., Neuromonitoring in Mild, Moderate, and Severe Acute Brain Trauma Using Non-invasive Diffuse Optics, p. 230, College of Medical and Dental Sciences, University of Birmingham (2021).

[74] Boas D. A. Dale A. M., “Simulation study of magnetic resonance imaging-guided cortically constrained diffuse optical tomography of human brain function,” Appl. Opt. 44(10), 1957–1968 (2005). APOPAI 0003-6935 10.1364/AO.44.001957 15813532

[75] Boas D. A. Dale A. M. Franceschini M. A., “Diffuse optical imaging of brain activation: approaches to optimizing image sensitivity, resolution, and accuracy,” NeuroImage 23 Suppl. 23, S275–S288 (2004). 10.1016/j.neuroimage.2004.07.011 15501097

[76] Clancy M. et al., “Monitoring the injured brain – registered, patient specific atlas models to improve accuracy of recovered brain saturation values,” Proc. SPIE 9538, 95381C (2015). PSISDG 0277-786X 10.1117/12.2183783

[77] Clancy M. et al., “Improving the quantitative accuracy of cerebral oxygen saturation in monitoring the injured brain using atlas based near infrared spectroscopy models,” J. Biophotonics 9(8), 812–826 (2016). 10.1002/jbio.201500302 27003677

[78] Clancy M., “Application and development of high-density functional near infrared spectroscopy for traumatic brain injury,” School of Chemistry, University of Birmingham, University of Birmingham UBIRA E THESES (2017).

[79] Gagnon L. et al., “Short separation channel location impacts the performance of short channel regression in NIRS,” NeuroImage 59(3), 2518–2528 (2012). NEIMEF 1053-8119 10.1016/j.neuroimage.2011.08.095 21945793 PMC3254723

[80] Scholkmann F. et al., “Absolute values of optical properties ($\mu_{a}$, $\mu_{s}$, $\mu_{\mathrm{eff}}$ and DPF) of human head tissue: dependence on head region and individual,” Adv. Exp. Med. Biol. 1072, 325–330 (2018). AEMBAP 0065-2598 10.1007/978-3-319-91287-5_52 30178366

[81] Coles J. P. et al., “Effect of hyperventilation on cerebral blood flow in traumatic head injury: clinical relevance and monitoring correlates,” Crit. Care Med. 30(9), 1950–1959 (2002). CCMDC7 0090-3493 10.1097/00003246-200209000-00002 12352026

[82] Baker W. B. et al., “Continuous non-invasive optical monitoring of cerebral blood flow and oxidative metabolism after acute brain injury,” J. Cereb. Blood Flow Metab. 39(8), 1469–1485 (2019). 10.1177/0271678X19846657 31088234 PMC6681541

[83] Perkins G. A. Eggebrecht A. T. Dehghani H., “Quantitative evaluation of frequency domain measurements in high density diffuse optical tomography,” J. Biomed. Opt. 26(5), 056001 (2021). JBOPFO 1083-3668 10.1117/1.JBO.26.5.056001 33949158 PMC8094378

[84] Perkins G. A. Eggebrecht A. Dehghani H., “Multi-modulated frequency domain high density diffuse optical tomography,” Biomed. Opt. Express 13, 5275–5294 (2022). BOEICL 2156-7085 10.1364/BOE.467614 36425621 PMC9664897

[85] https://www.gill-learning.co.uk.

